# Long-day photoperiod and cool temperature induce flowering in cassava: Expression of signaling genes

**DOI:** 10.3389/fpls.2022.973206

**Published:** 2022-09-16

**Authors:** Peter T. Hyde, Tim L. Setter

**Affiliations:** Section of Soil and Crop Sciences, School of Integrative Plant Science, Cornell University, Ithaca, NY, United States

**Keywords:** flower induction, flower initiation, photoperiod, temperature, transcriptome expression, RNA-seq, ambient temperature, *Manihot esculenta*

## Abstract

Cassava is a staple food crop in the tropics, and is of particular importance in Africa. Recent development of genomic selection technology have improved the speed of cassava breeding; however, cassava flower initiation and development remains a bottleneck. The objectives of the current studies were to elucidate the effect of photoperiod, temperature and their interactions on the time of flowering and flower development in controlled environments, and to use RNA-sequencing to identify transcriptome expression underlying these environmental responses. Compared to a normal tropical day-length of 12 h, increasing the photoperiod by 4 h or decreasing the air temperature from 34/31 to 22°/19°C (day/night) substantially hastened the time to flowering. For both photoperiod and temperature, the environment most favorable for flowering was opposite the one for storage root harvest index. There was a pronounced treatment interaction: at warm day-time temperatures, percent flowering was low, and photoperiod had little effect. In contrast, at cooler temperatures, percent flowering increased, and long-day (LD) photoperiod had a strong effect in hastening flowering. In response to temperature, many differentially expressed genes in the sugar, phase-change, and flowering-time-integrator pathways had expression/flowering patterns in the same direction as in Arabidopsis (positive or negative) even though the effect of temperature on flowering operates in the reverse direction in cassava compared to Arabidopsis. Three trehalose-6-phosphate-synthase-1 (TPS1) genes and four members of the SPL gene family had significantly increased expression at cool temperature, suggesting sugar signaling roles in flower induction. In response to LD photoperiod, regulatory genes were expressed as in Arabidopsis and other LD flowering plants. Several hormone-related genes were expressed in response to both photoperiod and temperature. In summary, these findings provide insight on photoperiod and temperature responses and underlying gene expression that may assist breeding programs to manipulate flowering for more rapid crop improvement.

## Introduction

Cassava (*Manihot esculenta*, Crantz) is a tropical crop grown as a source of food and specialty starch products. More than half of the worldwide production occurs in Africa where hundreds of millions of people depend on cassava as a staple food (Jarvis et al., 2012; Parmar et al., 2017). The multiple uses of cassava include food from the storage roots and leaves, tapioca and other processed starch products, and livestock feed (Parmar et al., 2017). The ability of cassava to produce appreciable yields under sub-optimal conditions has led to its wide adoption by both small- and large-holder farmers. Furthermore, with respect to climate change, given its relative tolerance of drought and high optimal temperature for growth, it is predicted to be one of the least adversely affected staple-food crops in sub-Saharan Africa (SSA) (Jarvis et al., 2012), further increasing the need for research and development of this vital crop.

Breeding is needed to develop cultivars for the diversity of farmer’s preferences including high yield, disease resistance and consumer-preferred quality traits. Village surveys have determined that small holder farmers grow multiple cultivars in regions such as Uganda (Iragaba et al., 2020) and Ghana (Rabbi et al., 2015). These different varieties serve multiple needs including risk aversion and market demands (Nakabonge et al., 2018). Furthermore, consumer-preferred traits vary by gender and locale (Teeken et al., 2018; Iragaba et al., 2021) such that including these varied traits in breeding schemes can improve adoption of improved varieties (Iragaba et al., 2021). Breeding for resistance to newly emerging diseases such as cassava brown streak disease (CBSD) is needed to reduce devastating outbreaks (Kawuki et al., 2016). These reports emphasize the need to breed a large diversity of improved cultivars that can be made available to farmers.

Recent developments of genomic selection technology have improved the speed of cassava breeding (Wolfe et al., 2017; Andrade et al., 2019). However, reliable and prompt flower initiation and development, which are essential for conventional breeding and genomic selection, remains a bottleneck (Alves, 2002; Ceballos et al., 2012; Andrade et al., 2019). The timing of floral initiation of the apical meristem is exceptionally variable, with some varieties first developing an inflorescence as early as 2 months after planting and others almost never produce an inflorescence (Ceballos et al., 2004; Adeyemo et al., 2019; Pineda et al., 2020a; Tokunaga et al., 2020). Even if a plant initiates an inflorescence it often produces few or no viable female flowers, further hindering breeding efforts (Adeyemo et al., 2019; Hyde et al., 2020; Pineda et al., 2020b).

Flowering in cassava has long been thought to be induced by long-day photoperiods (Keating et al., 1982; Pineda et al., 2020a; Souza et al., 2020; Tokunaga et al., 2020); however, these studies involved naturally occurring environmental conditions in the field where interpretation of the response to one environmental property is confounded by variation in several others. For example, Keating et al. (1982) planted cassava over several months in a location at 27°S latitude and observed that time-to-flowering was earlier in mid-summer, leading them to suggest that cassava flowers in response to long days, even though there were other co-variate factors, such as temperature, which were not evaluated. Similarly, studies that involved evaluating flowering over a 1-year time-frame have led researchers to conclude that cassava responds to long days (Souza et al., 2020). More recently, studies in controlled environments have shown that cassava flowering is indeed induced in long days, but also by cool temperatures (22°C) (Adeyemo et al., 2019; Oluwasanya D. N. et al., 2021).

In the model species Arabidopsis, signaling pathways that regulate the transition from vegetative growth phase to reproductive phase have been identified and characterized in detail. These pathways include circadian, hormone, autonomous, age, sugar, vernalization, ambient temperature, and photoperiod (Fornara et al., 2010; Blümel et al., 2015). There is a convergence of these signaling pathways toward a small number of floral integrator genes, notably FLOWERING LOCUS T (FT). FT encodes a phloem-mobile signaling protein which travels from the leaves to the apical meristem where it promotes floral induction. Several genes in the photoperiod pathway have been identified in cassava as homologs of their counterparts in Arabidopsis and other species, including MeFT1 and MeFT2, which are homologs of Arabidopsis FT (Adeyemo et al., 2019; Behnam et al., 2021). A large number of other photoperiod signaling homologs have been identified in cassava and their expression detected by transcriptome analysis, including TERMINAL FLOWER1 (TFL1), CONSTANS (CO), GIGANTEA (GI), and TEMPRANILLO1 (TEM1) (Adeyemo et al., 2019; Tokunaga et al., 2020; Behnam et al., 2021; Oluwasanya D. et al., 2021). Additionally, overexpression of Arabidopsis FT in cassava induces both flower initiation (Adeyemo et al., 2017; Bull et al., 2017; Odipio et al., 2020) and flower proliferation (Adeyemo et al., 2017). Together, this shows that many of the genes regulating flowering in model species have homologs in cassava and behave in a similar manner.

In the current study we elucidated the effect of photoperiod, temperature and their interactions on the time-to-flowering and flower development in cassava genotypes. To further our understanding of the mechanisms behind these environmental effects we investigated the transcriptome of plants grown under controlled environments of temperature and photoperiod. For several flowering regulatory pathways, we compared the transcriptome expression in cassava with that seen in Arabidopsis and other species, and obtained insight that may assist breeding programs in manipulating flowering for more rapid crop improvement.

## Materials and methods

### Plant material

Five cassava genotypes were used in the photoperiod and temperature experiments. TMS-IBA-980002 (also known as TMSI980002) and TMEB419 were obtained from the International Institute of Tropical Agriculture (IITA), Ibadan, Nigeria; Nase14, Nase3 (also known as TMS30572) and TME204 were obtained from the National Crop Resources Research Institute (NaCRRI), Namulonge, Uganda.

### Growth conditions

Stem cuttings (stakes) were cut to about 15 cm length from the bottom 1 m of previously grown plants. Stakes were planted into 11-L pots (Polytainer #3; Nursery Supplies Inc., Chambersburg, PA, United States). Rooting media was a mixture of peat:vermiculite:perlite (62:22:11; v:v) with added dolomitic limestone and 2.2% (w:v) of fertilizer (10-5-10 Jacks Pro Media mix plus III; J.R. Peters, Inc., Allentown, PA, United States), as previously described (Hyde et al., 2020).

### Photoperiod experiment 1 and 2

Two photoperiod experiments were conducted in a pair of matched growth chambers (Sherer, model CEL 511-38 walk-in room, 130 cm × 260 cm × 200 cm [depth × width × ht.], Sherer Inc., Marshall, MI, United States) with illumination by Philips cool white (4100 K) fluorescent lamps (Amsterdam, Netherlands) which provided 400 μmol m^–2^ s^–1^ (400–700 nm) photon flux density. In both Photoperiod Exp. 1 and 2, treatments were short day (SD), with 10 h of illumination from 6:00 until 16:00, and long day (LD) with 10 h of full illumination from 06:00 until 16:00 and with an additional 4 h of illumination with 10 μmol m^–2^ s^–1^ (dim light extension) from 16:00–20:00 provided by red light-emitting-diode (LED) lamps (spectral peak at 660 nm). In Photoperiod Exp. 1, temperature was 25°C (±0.38°C STD) starting at the beginning of the light period and extending 12 h, and 20°C for the second 12 h of a 24-h period. In Photoperiod Exp. 2, temperature was 30°C (±0.32°C SD) starting at the beginning of the light period and extending 12 h, and 25°C for the second 12 h of a 24-h period.

Each of the photoperiod experiments had two sequential batches of plants, each of which had 3 replicates of each genotype (TMSI980002, Nase 3, Nase 14 and TME 419) in a randomized block design, where batches (blocks) were considered a random effect. Photoperiod experiments were terminated when plants out-grew the height of the growth chamber; this averaged 182 d after planting (DAP).

### Photoperiod × temperature experiment

The Photoperiod × Temperature Experiment was conducted in four matched growth chambers (Model CEL-63-10, Sherer Inc., Marshall, MI, United States) which had interior dimension of 112 cm × 74 cm × 83 cm (width × depth × ht.) and 400 μmol photons of photosynthetically active radiation (400–700 nm) m^–2^ s^–1^ at the top of the canopy, supplied by fluorescent lamps (Philips F48T8/TL841/HO). The two temperature treatments were (1) warm, with 35°C (±0.18 SD) from 6:00 until 18:00 (day) and 30°C (±0.12) from 18:00 until 06:00 (night), and (2) cool, with 25°C (±0.22) from 6:00 until 18:00 (day) and 20°C (±0.13) from 18:00 until 06:00 (night). Photoperiod treatments were short day (illumination of 400 μmol m^–2^ s^–1^ from 6:00 until 18:00) and long day (illumination of 400 μmol m^–2^ s^–1^ from 6:00 until 18:00 with 10 μmol m^–2^ s^–1^ dim light extension from 18:00–22:00). Lighting for dim light extension was provided by Philips Decorative Twister (4100K) lamps. The experiment had a 2 × 2 factorial arrangement in a randomized complete block design of two temperatures and two photoperiods (four treatment combinations). The experiment was run in four sequential batches (blocks), each containing a complete representation of the four genotypes and four temperature × photoperiod treatments. Treatments where imposed for an average of 114 days and experiments ended when plants out-grew the height of the growth chambers.

### Temperature experiment

The Temperature Experiment was conducted in three matched growth chambers (Conviron Controlled Environments Ltd., Winnipeg, MB, Canada) (135 cm × 245 cm × 180 cm [depth × width × ht.]) with ten 400 W high pressure sodium and ten 400 W metal halide lamps, providing 600 μmol photons (400–700 nm) m^–2^ s^–1^ with a 12 h photoperiod. The daytime temperatures were 22, 28, and 34°C and night temperatures were 3°C lower than the day. Two sequential batches of plants were run, each including all three temperatures. While the purpose of the study was to elucidate temperature effects, we included a range of genotypes: the first batch had four replicate plants of TMSI980002 and three replicates of Nase 3, Nase 14 and TME 419, and the second batch had five replications of TMSI980002 and two replications of Nase 3, Nase 14 and TME 419. The study was an unbalanced randomized block (batches) design with blocks considered a random effect and temperature and genotype fixed effects.

### Root zone temperature experiment

The Root-zone Temperature Experiment was conducted in the chambers described above for the photoperiod experiments. Plants were grown with 12-h daylength and chamber temperature of 30/25°C (day/night). Four root-zone temperature treatments were imposed: 15, 20, 30, and 40°C. Root zone temperatures were constant throughout night and day, and were obtained by installing about 1 m of copper tubing (9.5 mm outside dia.), which was coiled four turns such that the coils were about four cm from the periphery of the pot. Pots were insulated with 6-mm thick reflective bubble wrap insulation (Everbuilt Double Reflective Insulation, Home Depot Product Authority, Atlanta, GA, United States). Water was pumped through the coils, with the water temperatures thermostatically regulated by circulating thermo-controllers (Allied, Model 900, Fisher Scientific, Pittsburg, PA, United States), and soil temperature at the center of the pot was monitored and adjusted as needed. The duration of the experiment was 180 d. The experiment was a factorial arrangement of two genotypes (TMSI980002 and Nase 14) and four root-zone temperatures; two batches with two replicate plants for each treatment combination were run.

### Gene expression in response to temperature

An additional set of plants grown in two growth chambers as described for the Temperature Experiment (above) was used to evaluate temperature effects on gene expression. The daytime temperature treatments were 22 and 34°C with night temperatures 3°C lower than the day. TMSI980002 and TMEB419 were used with 17 replicate plants at 22°C and 14 replicate plants at 34°C. Leaves were sampled as described below.

### Gene expression in response to photoperiod

Leaves were sampled for analysis of gene expression from genotype TMEB 419 and Nase14 from Photoperiod Exp. 1 (described above). The experiment had photoperiods of 10 h (SD) and 14 h (LD) and day/night temperatures of 25/20°C. As described above, the LD treatment had 10 h of light at full flux density, followed by 4 h of dim light. Three replicate plants were used for each treatment × genotype combination. Leaf tissue was sampled 15 min before the end of the photoperiod at 69, 104, and 132 DAP, as described below.

### Flower terminology and data collection

Flower induction in cassava occurs when the shoot apical meristem transitions to an inflorescence meristem, which is accompanied by the growth of two to four axillary buds directly below the inflorescence (Perera et al., 2013; Pineda et al., 2020a). Shoot growth from these buds forms a fork, which is indicative of the floral induction event. Subsequent transitions of the shoot apices on each of the fork branches to inflorescences is described as second tier forking. The identification of a developing fork was used to determine the timing of flowering. Although the inflorescences of cassava are technically (botanically) cyathia (Perera et al., 2013), we will refer to the entire structure of petal-like bracts and associated pistils or anthers/stamens as female or male flowers, respectively. We will refer to the entire reproductive stalk with multiple female and male flowers as an inflorescence.

Weekly counts of the number of flower buds greater than 2 mm diameter and mature flowers were used to calculate (1) the maximum number of flowers on a given week on an individual plant (maximum flower count), (2) the number of days that an individual plant had non-senesced flowers (flower retention), and (3) the sum of all the weekly flower counts (flower integral). At the final harvest, storage roots were counted, and above-ground and storage-root plant material was separated, dried and weighed.

### Statistical analysis

Each experiment had a randomized complete block design. Mixed-model ANOVA was used with the modeled fixed effects including treatment (photoperiod and/or temperature), genotype, and genotype by treatment interaction. Batches of plants (complete blocks with all treatments and genotypes of a given experiment represented) were modeled as random effects, which accounted for batch-to-batch variation when the experiment was repeated in the same set of growth chambers over time. Linear Models and ANOVA were calculated using the lm and anova function of the “stats” package conducted in R studio (R Core Team, 2017). The emmeans package (Lenth, 2019) was used for mean comparisons both pairwise with *t*-tests and with multiple tests using Tukey–Kramer honest significant difference tests. The time to flowering or termination of experiment and the proportion of plants that flowered during the experiment were analyzed using the Cox proportional hazard test (Cox, 1972) in the *R* package “Survival” (Therneau, 2015). The loess curve-smoothing regression function (span = 2, degree = 2) of the “stats” package (R Core Team, 2017) was used to calculate the matrix used for the 3-dimensional image of photoperiod × temperature × percent flowering.

### Analysis of gene expression with RNA-sequencing

#### Tissue sampling

Three leaf lobes were sampled from the youngest fully developed leaves on the upper nodes of plants of the temperature and photoperiod experiments from the youngest fully expanded, mature leaf on each plant. Samples were excised approximately 15 min prior to the dark period, enclosed in porous polyester tea bags and immediately submerged in liquid N_2_ and subsequently transferred to a −80°C freezer awaiting RNA extraction.

#### RNA extraction

Total RNA was extracted by a modified CTAB protocol, and purified on silica RNA columns as previously described (Oluwasanya D. et al., 2021). Samples were ground in a mortar and pestle chilled with liquid N_2_; about 0.5 g of the powder was vortexed for 5 min with 1 mL of extraction buffer containing 1% [w/v] CTAB detergent, 100 mM Tris-HCl [pH 8.0], 1.4 M NaCl, 20 mM EDTA, and 2% [v/v] 2-mercaptoethanol followed by 0.2 mL of chloroform; the suspension was mixed for 1 min, tubes were centrifuged and the top layer was moved to a new tube and 700 μL of a buffer containing 4 M guanidine thiocyanate, 10 mM MOPS (pH 6.7) and 500 μL of 100% ethanol (100%) was added and mixed. This mixture was applied to silica RNA columns (RNA mini spin column, Epoch Life Science, Missouri City, TX, United States), then washed sequentially with 750 μL each of 10 mM MOPS-HCl [pH 6.7] with 1 mM EDTA, containing 80% [v/v] ethanol, then 80% ethanol (twice), and to elute the RNA, 20 μL RNAase-free water. The RNA quality was evaluated with a gel system (TapeStation 2200, Agilent Technologies, Santa Clara, CA, United States).

#### 3′RNA sequencing

The 3′RNA-seq libraries were prepared from ∼500 ng total RNA at the Cornell Genomics facility^1^ using the Lexogen QuantSeq 3′ mRNA-Seq Library Prep Kit FWD for Illumina (Greenland, NH, United States). For each experiment (temperature and photoperiod), the pool was sequenced on one lane of an Illumina NextSeq500 sequencer using Illumina bcl2fastq2 software. Illumina adapters were removed from the de-multiplexed fastq files using Trimmomatic (version 0.36; Bolger et al., 2014). Poly-A tails and poly-G stretches of at least 10 bases in length were then removed using the BBDuk program in the package BBMap^2^ (version 37.50), keeping reads at least 18 bases in length after trimming. Poly-G stretches result from sequencing past the ends of short fragments (G = no signal). The trimmed reads were aligned to the *Manihot esculenta* genome assembly 520_v7 (Mesculenta_520_v7.fa^3^) using the STAR aligner (version 2.7.0f; Dobin et al., 2012) allowing a read to map in at most 10 locations -outFilterMultimapNmax 10 with at most 6% mismatches (-outFilterMismatchNoverLmax 0.06), while filtering out all non-canonical intron motifs (-outFilterIntronMotifs RemoveNoncanonicalUnannotated). For the STAR indexing step, the number of reads overlapping each gene in the forward strand were counted using HTSeq-count [version 0.6.1 (Anders et al., 2015)].

#### Differential expression analysis

Analysis of differential gene expression was accomplished using the DESeq2 package (Love et al., 2014), which adjusts *P*-values for multiple testing due to the large number of tests. Manihot esculenta genes and their homologs from Arabidopsis thaliana and functional annotations were sourced from Phytozome13 (Bredeson et al., 2016). Arabidopsis flowering time genes, their pathways and expected effects on flowering were acquired from the Flowering Interactive Database FLOR-ID (Bouché et al., 2015). A list of 498 Manihot esculenta genes (version 7.1) and their annotations was created by matching them in Phytozome13^4^ (accessed 2021.08.08) with corresponding flowering time genes from FLOR-ID database for Arabidopsis. Also, 30 cassava homologs of flowering genes that were not included in auto-annotation, were added to the list.

## Results

### Photoperiod and temperature effects on flowering age and abundance

A series of tests showed that both photoperiod and air temperature influence the timing of cassava flower initiation (Table 1). In experiment Photoperiod 1 (Table 1A), with 25/20°C, the extended photoperiod (10 + 4 h) treatment hastened the days-to-flower at flowering tier 1 for the four genotypes by 22 d and increased the percentage of plants that flowered during the experimental period to 75% in the 10 + 4-h treatment, compared to 33% in the 10-h treatment. In experiment Photoperiod 2 (Table 1B), extended photoperiod decreased the average days-to-flower by 44 d and increased the percentage flowering from 0 to 58%. These effects were statistically significant (*P* ≤ 0.05) according to a Cox Proportional Hazard model, which considers both the proportion of plants that flower and the time that passes before a flower initiation event occurs (Cox, 1972). Similar treatment effects were observed at flowering tier 2. Comparing across the two experiments, flowering was earlier when plants were grown at cooler temperatures of 25/20°C (Photoperiod 1) than at warmer temperatures of 30/25°C (Photoperiod 2). Genotypic differences were seen in terms of the magnitude of treatment effect, but not a crossover interaction (Supplementary Tables 1, 2).

**TABLE 1:** Response of cassava to varying photoperiods and temperatures on time-to-flower and per-cent of flowering, in the first and second tier.

						Tier 1			Tier 2			Whole plant		
Experiment (table section)	Air Temperature (Day/Night)	Photo-period (h)	Root Zone Temperature (constant)	No. Genotypes	No. Plants	Days to flowering or termination	Percent flowering	Cox Proportional Hazard^†^	Days to flowering or termination	Percent flowering	Cox Proportional Hazard	Dried Weight (g)^¶^	Number of Roots	HI^§^
**(A) Photoperiod-1**	**25/20°C**	**10**		**4**	**26**	**134**	**33%**	**a**	**169**	**8%**	**a**	**167 a**	**15 a**	**0.62 a**
		10 + 4*				**112**	**75%**	**b**	**145**	**58%**	**b**	**180 b**	**14 a**	**0.48 b**

**(B) Photoperiod-2**	**30/25°C**	**10**		**4**	**24**	**207**	**0%**	**a**	**207**	**0%**	**a**	**262 a**	**9 a**	**0.39 a**
		**10 + 4**				**163**	**58%**	**b**	**198**	**25%**	**b**	**234 a**	**7 a**	**0.27 b**

**(C) Temperature**	**22/19°C**	**12**		**4**	**56**	**91**	**96%**	**a**	**137**	**75%**	**a**	**271 a**	**13 a**	**0.32 a**
	**28/25°C**					**116**	**75%**	**a**	**145**	**63%**	**a**	**557 b**	**12 ab**	**0.43 b**
	**34/31°C**					**151**	**21%**	**b**	**169**	**4%**	**b**	**531 b**	**9 b**	**0.42 b**

**(D) Root Zone Temperature**	**25/20°C**	**12**	**20°C**	**2**	**8**	**91**	**100%**	**a**	**NA**			**204 a**	**16 a**	**0.40 a**
			**30°C**			**81**	**100%**	**a**				**333 b**	**11 b**	**0.65 b**

**(E) Photoperiod × Temperature**	**2520°C**	**12 + 4**		**4**	**64**	**112**	**100%**	**a**	**NA**			**68 a**	**12 a**	**0.46 a**
		**12**				**132**	**50%**	**b**				**69 a**	**13 a**	**0.47 a**
	**35/30°C**	**12 + 4**				**154**	**0%**	**c**				**127 b**	**10 b**	**0.64 b**
		**12**				**154**	**0%**	**c**				**128 b**	**10 ab**	**0.70 b**

Plant growth was evaluated on a whole plant basis including whole plant dried biomass (Dried Weight), number of storage roots (Roots), and harvest index (HI). Shown are averages across genotypes and summary data for the five experiments; further details on each genotype are shown in Supplementary Tables. *Photoperiod +4 indicates a photoperiod extension with 4 h of dim light ca. 10 μmol m^–2^ s^–1^. ^†^Treatments within individual experiments not labelled with the same letter are significantly different using the Cox Proportional Hazard test with Bonferroni correction for multiple comparisons. This test uses a model that considers both the time that passes before a flower initiation event occurs and the fraction of plants that flower within the period of observation. ^¶^Treatments within an individual experiment not connected by same letter are significantly different using the t test for paired comparisons and Tukey HSD for multiple comparisons. ^§^HI (harvest index) is calculated as the proportion of the whole plant dried biomass that is dried storage root.

In the Temperature Experiment (Table 1C), plants grown at the coolest temperature of 22/19°C (day/night) flowered earliest (91 DAP), and had the highest percentage of plants flowering during the observation period (96%). When grown at the moderate temperature of 28/25°C, the mean age of flowering and percent flowering was intermediate (116 DAP, 75%), and at the highest temperature of 34/31°C the average age of flowering was the latest (151 DAP) and only 21% of plants flowered. Treatment comparisons were statistically significant (*P* ≤ 0.05) between the two lower temperatures and the high temperature using the Cox Proportional Hazard model to evaluate days-to-flowering and percent flowering. Flowering at the second tier had a similar pattern: plants at the two lower temperatures (daytime: 22 and 28°C) flowered earlier and a higher percentage flowered than at 34°C. Different genotypes had different responses to temperature in terms of the magnitude of the effect; however, there was not a crossover interaction and all genotypes had earlier flowering and a greater percentage of flowering at lower temperatures (Supplementary Table 3).

In the root zone temperature experiment (Table 1D), varying the root zone temperature 5°C above or below the air temperature of 25°C did not significantly affect days-to-flower and percent of plants that flowered, indicating that temperature response is likely due to above-ground processes.

To evaluate photoperiod × temperature interaction, a study with two temperatures (25/20°C and 35/30°C; day/night) and two photoperiods (12 h daylength and 12 + 4 h) was conducted (Table 1E, Photoperiod × Temperature Exp.). At 35/30°C, flowering was not observed during the experimental period in either photoperiod. However, at 25/20°C, flowering occurred in both photoperiods; among them, long daylength (LD) induced earlier days-to-flower and a higher percent flowering compared to short daylength (SD).

**FIGURE 1:**
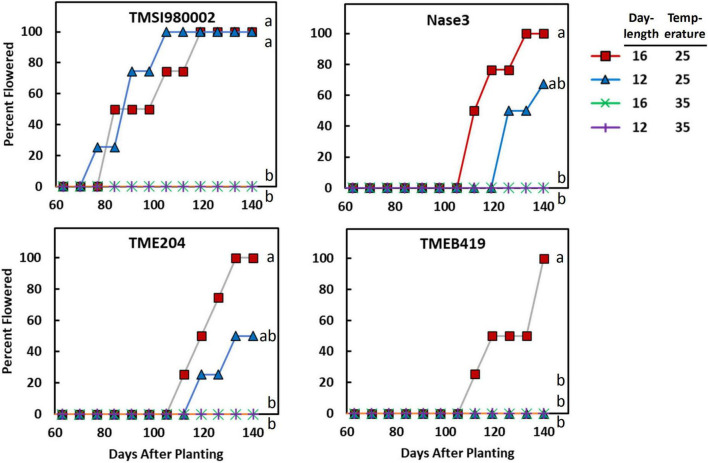
The effect of photoperiod and temperature on percentage of plants flowering as a function of time in the genotypes TMSI980002, Nase 3, TME 204, and TMEB 419. Day-lengths were 16 h (12 h full light + 4 h dim light) and 12 h full light. Temperatures were 25/20°C (day/night) and 35/30°C. Data shown represent the average of 4 replicate plants for each photoperiod × temperature × genotype combination. Curves labeled with different letters differed (*P* ≤ 0.05) according to Cox Proportional Hazard tests, which considers both the proportion of plants that flower and the time-to-flowering.

The response of each genotype to photoperiod × temperature indicated that in all genotypes except, TMSI980002, flowering was earliest at LD and cool temperature (Figure 1). Differences in flowering were indicative of a significant genotype by treatment interaction detected by ANOVA and Cox Proportional Hazard test (Supplementary Table 4). In TMSI980002, which began flowering much earlier than the others, plants in LD and SD flowered similarly at 25/20°C. In the genotype with latest flowering, TMEB419, flowering only occurred in LD and cool temperature.

Partitioning of carbon for the growth of alternative plant parts was also affected by photoperiod and temperature, but differently than flowering (Table 1, columns on right). At both 25/20 and 30/25°C, in Photoperiod Exp. 1 and 2, respectively, plants in the 10-h photoperiod were not significantly different from those in 14-h photoperiod in their total plant weight or root count; however, HI was significantly greater with 10-h daylength. When cassava was grown with 12-h daylength at three temperatures, both total plant dry weight and HI were higher at 28/25 and 34/31°C than at 22/19°C, though the number of storage roots were fewer at the warm temperatures. The photoperiod by temperature experiment also showed that when grown at 35/30°C, plants had a greater total weight and HI than plants grown at 25/20°C. Hence, for both photoperiod and temperature, the environment most favorable for flowering was opposite of the one for storage root HI: flowering was favored in LD and cool environments; whereas storage root HI was favored in SD and warm environments.

To determine the extent to which carbon partitioning responds to root temperature, we grew plants at a common above-ground temperature and subjected root-zones to two temperatures (Table 1D, Root Zone Exp.). At a warm root-zone temperature of 30°C compared to 20°C, flowering was not affected, while both total plant dry weight and storage root HI were higher at a root-zone temperature of 30°C than 20°C. Thus, whereas the temperature response of flowering was apparently due to above-ground temperature, carbon partitioning attributes were affected by both root-zone and whole-plant temperature.

**FIGURE 2:**
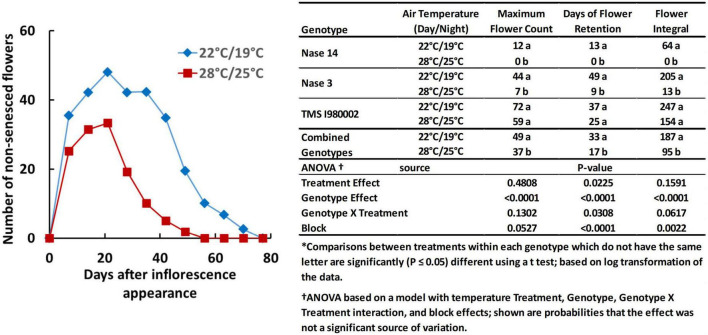
Floral development in genotypes Nase 14, Nase 3, and TMSI980002 at tier 2, grown at day/night temperatures of 22/19 and 28/25°C. Graph represents weekly mean flower counts averaged across all three genotypes.

Temperature also affected flower prolificacy, i.e., the number of flowers produced per plant, and inflorescence longevity, the time-frame over which flowers were produced and remained viable/non-senescent, as illustrated by the graph in Figure 2. For this experiment the late flowering lines (TME204 and TMEB419) were not included and an early line (Nase14) was substituted. At 22°C (day-time), the count number of non-senesced flowers averaged across the three genotypes reached a maximum of 49 flowers, whereas at 28°C, the average maximum was 37 flowers (Figure 2, embedded table). Longevity was also greater at the cooler temperature: at 22°C the average days of flower retention was 33 d, whereas at 28°C it was 17 d. The integral of flower counts over time (area under the curve) was 187 flower-days at 22°C and 95 flower-days at 28°C. Genotypes differed in these properties: In Nase14 and Nase 3, maximum flower counts, retention, and flowering integral were substantially greater at 22°C than 28°C (*P* < 0.05). In TMSI980002 flowering was abundant by all measures, though the effects of temperature on flowering were not significant (*P* ≤ 0.05).

### Gene expression in response to temperature and photoperiod

We conducted two studies to determine gene expression of the transcriptome in response to environmental factors: (1) a comparison of plants grown at cool temperatures (22/19°C day/night) vs. warm temperatures (34/31°C day/night), and (2) a comparison of plants grown in long-day (14 h) vs. short-day (10 h) photoperiods. In both cases, mature leaf tissue was sampled 15 min before the end of the photoperiod. Using an experiment-wise adjusted *P*-value (*P*_adj_) of 5% and genome-wide statistical analyses of differentially expressed genes, we identified 7946 genes that differed in the comparison of plants grown at 22 vs. 34°C (Supplementary Table 5), and 6616 genes that differed in the LD vs. SD comparison (Supplementary Table 6). To provide an overview of the types of genes that were affected by these environments, we performed enrichment analysis with ShinyGO (Ge et al., 2019) on each group of genes differentially expressed in response to treatments. This analysis identified gene ontology (GO) categories which were represented in greater proportion than what would be expected by random chance. In the temperature experiment, among genes that had significantly higher expression at 34°C than 22°C, genes upregulated by 34°C were enriched, based on the statistical false discovery rate (FDR) in the GO categories “response to stress” (FDR 1.4 × 10^–25^; 435 genes), “response to heat” (FDR 3.3 × 10^–17^; 61 genes) and “response to temperature stimulus” (FDR 5.2 × 10^–11^; 95 genes) (Supplementary Table 7). Collectively, among genes upregulated by 34°C, there were 593 genes in stress- and heat-related categories, whereas among genes downregulated by 34°C, there were only 102 genes. Hence, the high temperature treatment probably induced expression of a large number of genes not directly related the regulation of flowering, such as those associated with high temperature.

Enrichment analysis for the photoperiod experiment indicated that among genes downregulated in the LD treatment, GO categories related to photosynthesis and response to light were enriched relative to what could be attributed to random chance (Supplementary Table 7). In GO categories related to photosynthesis and response to light, there were 11 genes upregulated by LD relative to SD, and 166 downregulated in LD (i.e., upregulated in SD). This outcome is consistent with the light levels that existed when leaves were sampled for transcript analysis, which was about 15 min before the beginning of the dark period in each case. In the SD treatment, leaves were sampled at the end of the photosynthetic period when photon flux was high (400 μmol m^–2^ s^–1^) and photosynthesis was active. In the LD treatment, leaves were sampled during the dim-light extension of the photoperiod when photon flux was low (10 μmol m^–2^ s^–1^) and photosynthesis was minimal. Thus the expression profiles for both the temperature experiment and the photoperiod experiment likely included a large number of genes not directly related to regulation of flowering.

To assess expression of genes involved in flowering, we focused on cassava homologs of genes identified in the FLOR-ID database of flowering-related genes, a database that includes genes with both direct and indirect roles in signaling/regulating flower development (Bouché et al., 2015). From this database of Arabidopsis flowering-related genes, we obtained a list of 498 cassava homologs. Also, we added 30 cassava homologs of flowering-related genes that were not in the initial list (Supplementary Table 8). Among these genes, in the temperature experiment, plants grown at 22 vs. 34°C had 198 flowering-related differentially expressed genes (DEG) using an experiment-wise adjusted *P*-value of 5% (*P*_*adj*_ ≤ 0.05) (Supplementary Table 8). Based on the FLOR-ID database, we classified these genes into individual flower-signaling and regulatory pathways (Bouché et al., 2015).

#### Temperature

Several differentially expressed cassava genes (*P*_*adj*_ ≤ 0.05) that were categorized into the ambient temperature pathway were expressed at lower levels in cool than warm temperatures (Table 2). In cooler growth conditions, expression of cassava homologs of the Arabidopsis PIF family and FCA genes were decreased whereas SVP was increased. PIF-family genes are of particular interest because in Arabidopsis, PIF4 has a key role in the ambient temperature pathway and in regulation of flowering (Kumar et al., 2012; Proveniers and van Zanten, 2013). A comparison of amino acid sequences of the PIF homologs in cassava indicates that the cassava PIF that was significantly expressed in response to temperature, Manes.13G043000, had close similarity to Arabidopsis PIF4 and PIF3 (Table 2; Supplementary Figure 1). Given that cassava flowering was enhanced by cooler temperatures (Table 1), and that expression of cassava PIF4 and FCA homologs were significantly lower at cool temperature (Table 2), expression of these genes was negatively correlated with flowering. SVP was positively correlated with flowering. However, these expression patterns were opposite that found in Arabidopsis. While the temperature effects on expression were in the same direction in both species (lower expression at cool than warm temperature), in Arabidopsis warm temperature promotes flowering so expression of PIF and FCA is positively correlated with flowering and SVP expression is negatively correlated with flowering. Based on these findings we suggest that for these genes the mechanism by which temperature affects flowering in cassava differs from that in Arabidopsis, and may depend on additional factors or have opposite effects.

**TABLE 2:** Significant differentially expressed genes (DEGs) and log2 Fold Change of core downstream genes in the ambient temperature pathway.

Arabidopsis gene name	DESeq2 results: expression at 22 vs. 34°C		Correlation between gene expression and flowering			
	Log2 Fold Change	*P*_adj_	Cassava	Arabidopsis	*M. esculenta* Gene	*A. thaliana* Gene
**PIF**	**−1.34**	**0.0001**	**Negative**	**Positive**	**Manes.13G043000**	**AT2G20180.2**
**FCA**	**−0.60**	**0.0003**	**Negative**	**Positive**	**Manes.03G206500**	**AT4G16280.2**
**FCA**	**−0.54**	**0.0126**	**Negative**	**Positive**	**Manes.01G230100**	**AT4G16280.4**
**SVP**	**0.64**	**0.0074**	**Positive**	**Negative**	**Manes.10G099000**	**AT2G22540.1**

Several cassava genes which responded to temperature (*P*_adj_ ≤ 0.05) were classified into the sugar and aging pathways. The sugar pathway relates to the enhancement of flowering in response to abundant photosynthetic activity, while the “aging” pathway refers to developmental regulation of phase changes, notably the phase transitions from juvenile to adult and from vegetative to reproductive development. A high fraction of these genes (80% in sugar pathway and 100% in aging pathway) had correlations between gene expression and flowering that agreed with the direction of the response (positive vs. negative) in Arabidopsis (Table 3). Four genes in the sugar signaling and response pathway (TPS1, PGM1, SUS4, and ADG1) had higher expression in cassava plants grown at flower-enhancing 22 vs. 34°C. This is an expression pattern with the same relationship to flowering as in Arabidopsis (positive correlation). While several of the genes in this pathway could be viewed as enzymes whose role is metabolism rather than signaling (PGM1, SUS4, and ADG1), TPS1 (trehalose-6-phosphate synthase-1) is considered to have roles in signaling sugar status and regulating metabolic and developmental processes (Wahl et al., 2013; Ponnu et al., 2020). A comparison of amino acid sequences of the TPS1 homologs in cassava indicates that the cassava TPS1 genes that were significantly expressed in response to temperature (Manes.15G116000, Manes.15G098800, and Manes.17G062500), had close similarity to Arabidopsis TPS1 (Table 3; Supplementary Figure 2).

**TABLE 3:** Significant differentially expressed genes (DEGs) and log2 Fold Change from flowering pathways showing the most similarity to flower regulation in A thaliana and relevant down stream genes part of the flower development and meristem identity and flowering time integrator pathways.

		DESeq2 Results: expression at 22 vs. 34°C		Correlation between gene expression and flowering			
Pathway	Arabidopsis gene name	log2 Fold Change	Padj	Cassava	Arabidopsis	*M. esculenta* Gene	*A. thaliana* Gene
**Sugar**	**ADG1, APS1**	**1.03**	**<0.0001**	**Positive**	**Positive**	**Manes.12G067900**	**AT5G48300.1**
	**ADG1, APS1**	**1.22**	**<0.0001**	**Positive**	**Positive**	**Manes.13G058900**	**AT5G48300.1**
	**AKIN10, SNRK1.1**	**0.42**	**0.0104**	**Positive**	**Negative**	**Manes.02G049300**	**AT3G01090.2**
	**HXK1, GIN2**	**−1.93**	**<0.0001**	**Negative**	**Positive**	**Manes.03G026700**	**AT4G29130.1**
	**PGM1**	**0.82**	**0.0094**	**Positive**	**Positive**	**Manes.06G141300**	**AT5G51820.1**
	**PGM1**	**0.67**	**0.0795**	**Positive**	**Positive**	**Manes.14G031100**	**AT5G51820.1**
	**SUS4**	**0.98**	**<0.0001**	**Positive**	**Positive**	**Manes.03G044400**	**AT3G43190.1**
	**SUS4**	**1.05**	**<0.0001**	**Positive**	**Positive**	**Manes.16G090600**	**AT3G43190.1**
	**TPS1**	**2.95**	**<0.0001**	**Positive**	**Positive**	**Manes.15G116000**	**AT1G78580.1**
	**TPS1**	**0.54**	**0.0003**	**Positive**	**Positive**	**Manes.15G098800**	**AT1G78580.1**
	**TPS1**	**1.63**	**0.0381**	**Positive**	**Positive**	**Manes.17G062500**	**AT1G78580.1**

**Developmental Age**	**SPL3**	**1.34**	**<0.0001**	**Positive**	**Positive**	**Manes.17G047500**	**AT2G33810.1**
	**SPL3**	**2.41**	**<0.0001**	**Positive**	**Positive**	**Manes.16G029900**	**AT2G33810.1**
	**SPL5**	**1.28**	**0.0118**	**Positive**	**Positive**	**Manes.03G106900**	**AT3G15270.1**
	**SPL9**	**1.80**	**<0.0001**	**Positive**	**Positive**	**Manes.09G032800**	**AT2G42200.1**
	**TOE1, RAP2.7**	**−1.06**	**0.0001**	**Negative**	**Negative**	**Manes.10G041100**	**AT2G28550.3**
	**TPL**	**−0.52**	**<0.0001**	**Negative**	**Negative**	**Manes.09G124300**	**AT1G15750.1**
	**TPL**	**−1.83**	**<0.0001**	**Negative**	**Negative**	**Manes.16G124800**	**AT1G15750.3**
	**TPL**	**−0.64**	**0.0131**	**Negative**	**Negative**	**Manes.04G108600**	**AT1G15750.4**
	**TPL**	**−0.27**	**0.0829**	**Negative**	**Negative**	**Manes.08G164000**	**AT1G15750.4**

**Flower development and meristem identity**	**AP2**	**−0.43**	**0.1422**	**Negative**	**Negative**	**Manes.12G106400**	**AT4G36920.1**
	**FUL, AGL8**	**1.67**	**<0.0001**	**Positive**	**Positive**	**Manes.14G088500**	**AT5G60910.1**
	**FUL, AGL8**	**0.70**	**0.0064**	**Positive**	**Positive**	**Manes.02G059300**	**AT5G60910.1**

**Flowering time integrator**	**SOC1, AGL20**	**0.29**	**0.1225**	**Positive**	**Positive**	**Manes.01G263500**	**AT2G45660.1**
	**SOC1, AGL20**	**0.22**	**0.4651**	**Positive**	**Positive**	**Manes.05G041900**	**AT2G45660.1**

The reference condition was 34°C.

In the developmental age pathway (regulation of developmental phase transitions), SPL genes (SPL3, SPL5, and SPL9) had higher expression in cassava plants which had enhanced flowering at 22°C (Table 3), matching their positive correlation with flowering in Arabidopsis. Also, in the aging pathway four different TPL homologs, and one TOE1 homolog had expression that matched the Arabidopsis direction of relationship to flowering, though in this case the magnitude of expression was negatively correlated with tendency for flowering at 22°C. Furthermore, three flower development and identity DEGs homologous with FUL and AP2, and two homologs of the flower time integrator gene, SOC1, were expressed in the same manner as Arabidopsis. These findings indicate that many of the DEGs in the sugar, aging, and flowering-time-integrator pathways had expression/flowering patterns in the same direction as in Arabidopsis (positive or negative) even though the effect of temperature on flowering operates in the reverse direction in cassava compared to its direction in Arabidopsis.

#### Photoperiod

To evaluate transcript expression in response to photoperiod, we compared plants grown in a short-day (SD) 10 h photoperiod vs. in a long-day (LD) extended photoperiod (10 h + 4 h). The sampling time was about 15 min before the dark period in each case, thus targeting differential expression caused by photoperiod signaling systems. Due to this sampling method, we have avoided placing our focus on genes whose expression is strictly related to time-of-day and the circadian cycle, and instead have focused on photoperiod-related genes. Two phytochrome genes PHYA and PHYB were both differentially expressed between long and short days (Table 4). PHYA was upregulated in long days whereas PHYB was down regulated, which indicates PHYA expression was positively correlated with flowering whereas PHYB was negatively correlated with flowering. These findings in cassava match the relationship of these genes to flowering in Arabidopsis. Two genes belonging to the family of phytochrome interacting factors (PIF) were also up-regulated by long days, matching the positive relationship between gene expression and flowering that is found in Arabidopsis. The cryptochromes CRY1 and CRY2 also had higher expression in long days, with a relationship between expression and flowering that matched that in Arabidopsis, and two negative factors in the cryptochrome pathway, COP1 and SPA1, were expressed at lower levels in LD, a pattern that is in the same direction as in Arabidopsis. Genes downstream of the phytochrome and cryptochrome pathways, in the CONSTANS-like (COL) gene family, also had expression in the same positive direction in cassava and Arabidopsis. Three members of the FBH family of CONSTANS-interacting factors had significant differential expression in response to photoperiod, and two of these had expression with a positive correlation with flowering, as in Arabidopsis, while one was negative.

**TABLE 4:** Significant differentially expressed genes (DEGs) and log2 Fold Change from flowering pathways showing the most similarity to flower regulation in A thaliana and relevant down stream genes part of the phytochrome and cryptochrome light signaling pathways.

		DESeq2 Results: expression at Long-day vs. Short-day		Correlation between gene expression and flowering			
Pathway	Arabidopsis gene name	log2 Fold Change	Padj	Cassava	Arabidopsis	*M. esculenta* Gene	*A. thaliana* Gene
**Phytochrome**	**PHYA**	**0.41**	**<0.0001**	**Positive**	**Positive**	**Manes.09G182500**	**AT1G09570.1**
	**PHYB**	**−0.48**	**<0.0001**	**Negative**	**Negative**	**Manes.03G205100**	**AT2G18790.1**
	**PIF**	**0.64**	**0.0008**	**Positive**	**Positive**	**Manes.12G044000**	**AT2G20180.3**
	**PIF**	**0.70**	**0.0191**	**Positive**	**Positive**	**Manes.13G043000**	**AT2G20180.2**
	**ATCOL2,COL2**	**2.11**	**<0.0001**	**Positive**	**Positive**	**Manes.01G106200**	**AT3G02380.1**
	**ATCOL2,COL2**	**1.54**	**<0.0001**	**Positive**	**Positive**	**Manes.02G062700**	**AT3G02380.1**
	**FBH3, AKS1, BHLH122**	**0.96**	**0.0001**	**Positive**	**Positive**	**Manes.06G167700**	**AT1G51140.1**
	**FBH4, AKS3**	**1.69**	**<0.0001**	**Positive**	**Positive**	**Manes.10G146400**	**AT2G42280.1**
	**FBH4, AKS3**	**−0.44**	**0.0010**	**Negative**	**Positive**	**Manes.09G031000**	**AT2G42280.1**

**Crypto-chrome**	**CRY1**	**0.84**	**<0.0001**	**Positive**	**Positive**	**Manes.02G152400**	**AT4G08920.1**
	**CRY1**	**0.48**	**<0.0001**	**Positive**	**Positive**	**Manes.18G067300**	**AT4G08920.1**
	**CRY2**	**0.31**	**0.0115**	**Positive**	**Positive**	**Manes.15G040500**	**AT1G04400.1**
	**COP1**	**−0.21**	**0.0108**	**Negative**	**Negative**	**Manes.12G068900**	**AT2G32950.1**
	**SPA1**	**−0.98**	**<0.0001**	**Negative**	**Negative**	**Manes.01G248600**	**AT2G46340.1**

Short-day was the reference condition.

#### Significant differentially expressed genes found in both the temperature and photoperiod experiments

Given that both LD photoperiod and cool temperature induced flowering, we explored which genes were differentially expressed in response to both treatments. Eighteen differentially expressed genes were identified as significant in both the photoperiod and temperature experiments (Table 5). As expected, many of these genes appear to be in pathways downstream of initial environmental perception at points that integrate multiple signaling pathways to determine flowering time. For example, genes in the pathways for aging (developmental phase change), CONSTANS-like (COL), flower development, and meristem identity were among the genes that were significantly up-regulated in response to both LD and cool temperature. A circadian clock LUX homolog was down-regulated in response to both flower-inducing treatments. There were also several genes in various general pathways, and some related to hormone signaling which were significantly affected by both photoperiod and temperature. Among the hormone-related genes were those involving gibberellins (GID1C, GA2), cytokinin (RR2), and auxin (IAA7). These findings indicate that there are numerous regulatory pathways that operate in response to both environmental factors, thus helping us distinguish pathways that may interact or are additive from those that may operate differently in each environmental response.

**TABLE 5:** Significant differentially expressed genes (DEGs) and log2 Fold Change from flowering pathways that where identified in both photoperiod and temperature induction of flowering in cassava.

		DESeq2 Results: expression at Long-day vs. Short-day		DESeq2 Results: expression at 22 vs. 34°C			
Pathway	Arabidopsis gene name	log2 Fold Change	*P*_adj_	log2 Fold Change	*P*_adj_	*M. esculenta* Gene	*A. thaliana* Gene
**Aging**	**SPL3**	**0.71**	**<0.0001**	**1.34**	**<0.0001**	**Manes.17G047500**	**AT2G33810.1**
**Circadian Clock**	**LUX, PCL1**	**−1.12**	**<0.0001**	**−1.08**	**<0.0001**	**Manes.01G170000**	**AT3G46640.1**
**Flower development and meristem identity**	**FUL, AGL8**	**1.06**	**<0.0001**	**1.67**	**<0.0001**	**Manes.14G088500**	**AT5G60910.1**
	**AGL1,SHP1**	**0.76**	**<0.0001**	**2.46**	**<0.0001**	**Manes.02G085501**	**AT3G58780.1**
	**AGL4,SEP2**	**0.76**	**0.0028**	**1.23**	**0.0009**	**Manes.01G103100**	**AT3G02310.1**
	**AGL1,SHP1**	**0.41**	**0.0321**	**2.21**	**<0.0001**	**Manes.01G128500**	**AT3G58780.1**

**General**	**MRG1**	**−0.54**	**0.0166**	**−1.36**	**<0.0001**	**Manes.05G178100**	**AT4G37280.1**
	**OTS1, ULP1D**	**−0.33**	**0.0753**	**−0.51**	**0.0169**	**Manes.12G029500**	**AT1G60220.1**
	**HTA9**	**−0.95**	**<0.0001**	**−0.21**	**0.0924**	**Manes.03G018100**	**AT1G52740.1**
	**UBC2**	**0.28**	**0.0027**	**0.50**	**0.0107**	**Manes.08G154200**	**AT2G02760.1**
	**MYB30**	**0.45**	**0.0177**	**0.80**	**0.0020**	**Manes.09G135700**	**AT3G28910.1**

**Hormones**	**GID1C**	**−1.07**	**<0.0001**	**−0.62**	**0.0096**	**Manes.09G161600**	**AT5G27320.1**
	**RR2, ARR2**	**−1.69**	**0.0039**	**−1.38**	**0.0279**	**Manes.01G262600**	**AT4G16110.1**
	**GA2, ATKS1**	**−0.81**	**0.0751**	**−2.88**	**<0.0001**	**Manes.16G068951**	**AT1G79460.1**
	**MYB33**	**0.89**	**<0.0001**	**1.23**	**0.0007**	**Manes.11G009900**	**AT5G06100.2**
	**IAA7, AXR2**	**0.48**	**0.0038**	**1.15**	**<0.0001**	**Manes.03G169700**	**AT3G23050.1**

**Photoperiodism, light perception and signaling**	**ATCOL2,COL2**	**2.11**	**<0.0001**	**1.31**	**<0.0001**	**Manes.01G106200**	**AT3G02380.1**
	**ATCOL2,COL2**	**1.54**	**<0.0001**	**1.16**	**<0.0001**	**Manes.02G062700**	**AT3G02380.1**

## Discussion

### Effect of temperature and photoperiod on flower induction

The current studies were conducted with the overarching goal of facilitating cassava breeding of genotypes which are poor flowering but have desirable agronomic traits. To this end, we evaluated the effect of photoperiod and temperature, two environmental factors that are known to affect flowering in many flowering plant species (Yan and Wallace, 1996). Our results show that compared to a normal tropical day-length of 12 h, increasing the photoperiod by 4 h decreased the time to flowering and increased the percentage of plants that flowered. Decreasing the air temperature from 34/31 to 22/19°C (day/night) greatly hastened the time to flowering on both the first and second tier of plant branching/flowering.

**FIGURE 3:**
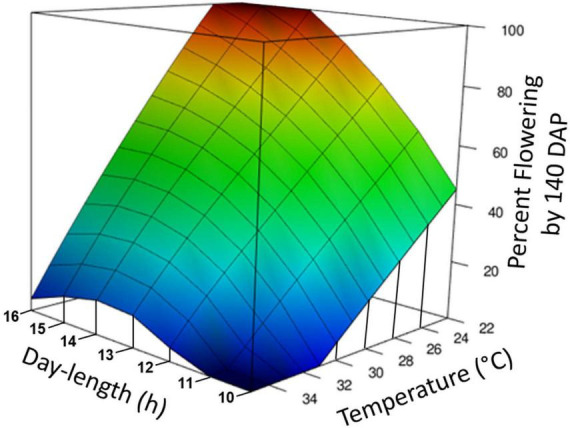
Graphical representation of photoperiod and temperature effect on percentage of plants induced to flower. The response surface was calculated by loess regression based on data from experiments Photoperiod 1, Photoperiod 2, Temperature, and Photoperiod × Temperature. All experiments were standardized by using the data for percent forking at 140 days after planting (DAP) and genotypes TMS98I0002, Nase3, and TMEB419. Day-time temperatures are shown.

Temperature and photoperiod effects interacted (Table 1E). At the warm temperature of 35°C, extended photoperiod did not hasten flowering, whereas at cooler temperatures, genotypes responded to photoperiod and flowering was hastened by LD. A 3-dimensional summary of the response of percent flowering to photoperiod × temperature is shown in Figure 3. The response surface was calculated based on data from the all of the photoperiod and temperature experiments shown in Table 1. The response surface indicates that at warm day-time temperatures, percent flowering was low, and photoperiod had little effect. In contrast, at cooler temperatures, percent flowering increased, and a pronounced interaction with photoperiod induced the highest percent flowering with the combination of LD and cool temperatures.

Previous anecdotal evidence suggests that extended photoperiod is more effective at cooler temperatures (Pineda et al., 2020a), and previous growth chamber trials indicated cool temperature is favorable for flowering (Adeyemo et al., 2019; Oluwasanya D. N. et al., 2021). Our results substantiate that cassava breeders aiming to utilize extended photoperiod to produce more flowers would benefit from utilizing locations with cooler temperatures.

### Temperature effect on transcriptome

Due to global climate change, interest in understanding temperature effects on all aspects of plant development including flowering has increased (Lee et al., 2013; Jin and Ahn, 2021). Recent work in Arabidopsis has elucidated several regulatory factors by which temperature affects time-to-flowering (Capovilla et al., 2014; Susila et al., 2018; Jin and Ahn, 2021). However, in contrast to our findings in cassava where flowering was enhanced by cooler temperature (Table 1 and Figures 1, 3), in Arabidopsis, flowering is promoted by warmer temperature. Accordingly, in Arabidopsis, expression of the transcription factor PIF4 is increased at warmer temperatures and it binds to the promoter region of the key flower-inducing gene FT, thereby increasing its expression (Kumar et al., 2012). In the current work, expression of FT was below the detectable threshold of our RNA-seq method, so it was not possible to confirm that FT expression was higher at cool temperatures, as would be expected from our flowering results. Our findings indicate that expression of a member of the PIF family in cassava with close homology with AtPIF3 and AtPIF4 was also increased by warmer temperature (Table 2 and Supplementary Figure 2); however, warmer temperature decreased flowering in cassava. It is plausible that the cassava PIF homolog that was up-regulated at warm temperature, Manes.13G043000, is operating similarly to Arabidopsis PIF3 which has been shown to inhibit flowering, and knockdown of its expression results in earlier flowering (Oda et al., 2004). Alternatively, it is possible that other factors are operational in cassava’s response to temperature.

Other potential contributors to ambient temperature response are the transcription factor SVP, which in Arabidopsis interacts with the FT promoter and negatively regulates flowering (Lee et al., 2013), and FCA, which promotes flowering at higher temperatures through the induction of FT (Jung et al., 2012). In cassava these components of the thermosensory pathway were differentially expressed in response to temperature; however, their expression was correlated with temperature effects on flowering in the opposite direction (Table 2). In cassava flowering was enhanced at low temperature whereas in Arabidopsis flowering is enhanced by warmer temperature. These results indicate that these components of the ambient temperature pathway may operate differently in cassava, or interact with additional components.

**FIGURE 4:**
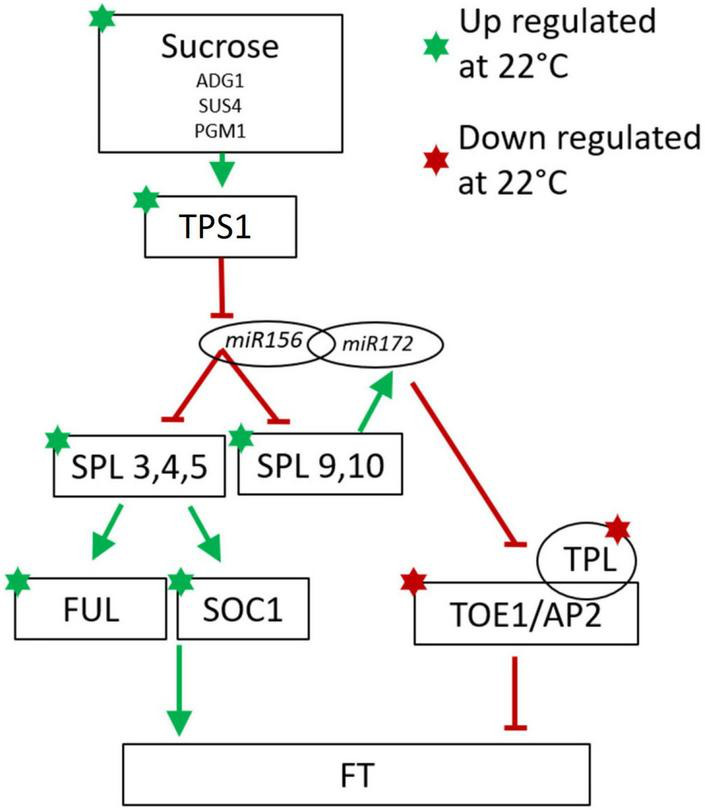
Minimal model of the of Arabidopsis sugar signaling and developmental phase change pathway for regulation of flowering with current findings for cassava gene expression indicated with stars for genes significantly up-(green) or down-(red) regulated at cool temperatures. Involvement of the non-coding microRNAs miR156 and miR172 are also shown. Arrows indicate promotive effects; transverse bars indicate inhibitory effects. Model adapted from: Wu et al. (2009), Wahl et al. (2013), Wang (2014), Ponnu et al. (2020).

In contrast to the lack of agreement between cassava and Arabidopsis in the correlation between temperature-regulated expression of the genes described in Table 2, a high proportion of flowering-related genes in the age signaling pathway (developmental phase change) and sugar signaling/response pathway were regulated in a direction similar to that seen in the model species Arabidopsis (Table 3). Phase change from juvenile to adult is needed for competence to flower (Poethig et al., 2013). A minimal model of these modules in Arabidopsis are represented in Figure 4 with the expression direction of homologs from the current study overlayed to assist in interpreting the differential expression we have found in cassava (Wu et al., 2009; Huijser and Schmid, 2011).

Consistent with the model in Figure 4, in cassava many of the genes in the SPL family including SPL3, 4, and 5, which promote flowering, and SPL 9 which promotes the juvenile to adult phase transition, had higher expression in when grown under cool temperatures which also induced flowering. In Arabidopsis, SPL9 interacts with miR172 to block the floral repressors TOE1 and AP2, and their activator, TPL (Wu et al., 2009; Huijser and Schmid, 2011; Wang, 2014). Consistent with their expected effects on flowering, in cassava, expression of genes homologous to TOE1, AP2 and TPL was significantly decreased when grown under cool flower inductive conditions.

SPLs in Arabidopsis are direct regulators of SOC1 and FUL which influence FT expression in the leaves (Wang et al., 2009; Yamaguchi et al., 2009; Wang, 2014). Our transcriptome analysis shows a concurrent up regulation of SPL3, SPL4, SPL5, SOC1, and FUL in the same manner as seen in Arabidopsis. The amino acid similarity of cassava homologs of the Arabidopsis SPL family are shown in Supplementary Figure 3. It has been shown in some woody perennial species including Citrus and Jatropha, that homologs of SPL3 and SPL5 are up-regulated in relation to flower initiation (Shalom et al., 2015; Yu et al., 2020). In Jatropha, a close relative of cassava, 15 SPL homologs were identified with most being highly conserved (Yu et al., 2020). Nine of the Jatropha SPL genes are regulated by miR156 (Yu et al., 2020). Jatropha JcSPL3 has increased expression in the leaves of plants entering the flowering stage of development and expression of JcSPL3 in Arabidopsis triggers earlier flowering (Yu et al., 2020). Together, based on this evidence, we suggest that cassava may operate similarly to Jatropha where JcSPL3 is responsible for vegetative phase transition (Yu et al., 2020).

In Arabidopsis many other signaling molecules are known to interact with the miR/SPL module. Trehalose-6-phosphate (T6P) signals carbohydrate status of the plant to regulate flowering (Wahl et al., 2013) and T6P-synthase-1 (TPS1) activity in the leaves is necessary for induction of FT. Recent work has shown that T6P regulates the juvenile-to-adult vegetative phase change by interactions with miR156/SPL module (Ponnu et al., 2020). In cassava, a high proportion of genes in the sugar-related pathway were expressed in response to temperature with the same relationship to flowering (positive vs. negative) as seen to induce flowering in Arabidopsis (Table 3). Among these are 3 TPS1 homologs (Table 3 and Figure 4). Based on this evidence, we suggest that in cassava, these genes might be involved in signaling carbohydrate status of the leaves to regulate flowering.

Several other regulatory networks have been shown to interact with the SPL/miR172 module and affect the time to flowering, including those involving the plant hormone GA, GI (the core regulatory component of the circadian pathway) (Wang, 2014) and TEMs (Aguilar-Jaramillo et al., 2019). In both field and greenhouse experiments involving temperature, Oluwasanya D. N. et al. (2021) found that expression of TEM2 in cassava was consistent with a role in regulating flowering. Our results did not show significant differentially expressed genes related to these pathways but they cannot be ruled out.

### Photoperiod effect on transcriptome

The external coincidence model for photoperiod regulation of flowering has many layers (Song et al., 2015). In general, our findings in cassava indicate that expression of genes in cassava follows this model, though as in other species, there may be some variation in these pathways (Hayama and Coupland, 2003; Song et al., 2015). As predicted by the model, whereby the circadian clock entrains accumulation of CONSTANS transcripts in the evening, in cassava, we found that two CO-like genes have significantly higher expression in the end-of-day (pre-dark) period in LD compared to SD (Table 4). The model predicts that this accumulation is followed by multiple layers of posttranslational regulation of the CO protein. PHYA stabilizes the CO protein (Hayama and Coupland, 2003; Song et al., 2015) and PHYB promotes the degradation of the CO protein (Hayama and Coupland, 2003). Blue light stabilizes CO protein through CRY and COP1. COP1 is a negative regulator of flowering, reducing CO abundance, and CRY negatively regulates COP1 (Liu et al., 2008). Our findings in cassava show evidence that expression of homologs for all of these components follow the expression predicted by the model (Table 4).

Studies of cassava conducted by Behnam et al. (2021) indicated that CO-like homologs COL2, COL3, and COL4 were expressed in leaves at young stages of plant development, whereas COL5, COL6, and COL7 were expressed at mature stages from 4 months after transplanting when flowering was taking place. These studies involved sampling at the middle of the day when CO expression might not be fully reflective of photoperiod or other environmental regulatory effects. Our studies, which involved sampling in the last 15 min of the light period, only found COL2 as differentially expressed in response to temperature and photoperiod (Tables 4, 5). Hence it is possible that only this homolog is involved in end-of-day expression associated with flower induction by these treatments, or differential expression of other CO homologs was below our limit of detection.

Other CO transcriptional promotors are also known to regulate the amplitude of CO transcripts in addition to the circadian cycling of expression. One such family are FBH1, 2,3,4 basic helix-loop-helix type transcription factors which bind directly to the CO promotor and likely function with multiple redundancies (Ito et al., 2012). In our study of cassava, two members of this family had higher expression in LD than SD, consistent with Arabidopsis, whereas a third member had the opposite pattern of expression in LD vs. SD (Table 4). The core circadian pathway gene GI, which has a strong circadian cyclical expression pattern that decreases in the evening (James et al., 2008; Bouché et al., 2015), was expressed at lower levels in long day plants sampled later in the evening, as expected (Supplementary Table 8). Behnam et al. (2021), sampling leaves at mid-day, found that in cassava GI followed the same pattern as FT with both genes expressed at low levels in leaves of young plants but higher in plants at the flowering stage of 4 months or older. Overall, the photoperiod regulatory system in cassava, in which flowering is promoted in long days, appears similar to the photoperiod regulatory mechanism in other long day flowering plants such as Arabidopsis.

### Temperature and photoperiod effect on transcriptome

Photoperiod and temperature elicited many of the same differentially expressed genes (Table 5). Some of these genes, such as CO and SPL may represent nodes that are targets of multiple upstream signaling pathways. These may help explain the interaction observed on the flowering response with the combination of LD and cool temperature (Figures 1, 3). Others such as flower development and identity genes may represent downstream effects that are part of later flower development. Because they have been identified to respond to both photoperiod and temperature it is likely they play a critical role in the induction of flowering, though at this point we do not have enough information to elucidate their exact function. Of particular interest are several hormone-related genes that were differentially expressed in response to both temperature and photoperiod treatments. In previous study, plant growth regulator (PGR) treatments with the anti-ethylene silver thiosulfate (STS) and cytokinin were found to be effective tools in stimulating flower proliferation and feminization of flower development in cassava (Hyde et al., 2020; Oluwasanya D. et al., 2021) and cytokinin stimulates female flower proliferation in Jatropha (Chen et al., 2014, 2019; Froeschle et al., 2017). A previous study of the effect of temperature on flowering in cassava identified differentially expressed genes in several hormone pathways (Oluwasanya D. N. et al., 2021). Collectively, these studies have revealed that application of these hormones stimulates expression in a wide range hormone pathways. It may be possible to use this information to further improve PGR treatment protocols or breeding efforts to improve flowering.

## Conclusion

### Environmental effects on flowering

These investigations showed that cooler air temperature and extended photoperiod stimulate earlier flowering, whereas these conditions were unfavorable to storage-root growth and harvest index. Warmer temperatures limited the benefit of extended photoperiod. Considering this, we conclude that the most favorable conditions for stimulating earlier and more prolific flowering in cassava are long-day photoperiods with relatively cool day temperatures of approximately 22°C.

Transcriptome data for cassava suggested that the regulatory system for photoperiod and downstream pathways leading to flower induction operate similarly to those in Arabidopsis. Many of the known genes involved in photoperiodism regulating flowering were differentially expressed in the same direction as in Arabidopsis, a model LD plant, confirming that cassava is also a long day plant. In contrast, Arabidopsis and cassava respond to ambient temperature in opposite directions –Arabidopsis flowers earlier in warm temperature whereas cassava flowers earlier in cool temperatures – yet expression of temperature-responsive flowering genes was similarly affected by temperature. These results indicate that the ambient temperature regulatory pathway as described based on studies of Arabidopsis does not function similarly in cassava flower induction. The sugar and developmental age pathways in Arabidopsis and cassava have similar gene expression patterns and correlation to flowering, therefore, it is likely these pathways are involved in the induction of flowering in cassava.

### Implication for breeding

The current findings provide an improved understanding of cassava flowering in response to photoperiod and temperature. Cassava breeders can utilize this information to provide guidance on the extent to which there may be benefit in locating their crossing nurseries where the climate is relatively cool and employing lights to extend the photoperiod. This is of immediate interest as global climate change is likely to increase average temperatures. This will be useful for all types of breeding designs that require making crosses, from genomic to mass selection approaches. Furthermore, breeders interested in developing non-branching cultivars can utilize this transcriptome information to target genes that may regulate flowering and in turn branching.

## Data availability statement

The original contributions presented in this study are publicly available. This data can be found here: https://cassavabase.org/ftp/manuscripts/Hyde_et_al_2022/.

## Author contributions

PH conducted the plant growth and laboratory work. PH and TS supervised the work, analyzed the data, wrote the manuscript, revised the article, and approved the submitted version. TS conceived the project and obtained funding. Both authors contributed to the article and approved the submitted version.

## Footnotes

1
http://www.biotech.cornell.edu/brc/genomics-facility

2
https://sourceforge.net/projects/bbmap/

3
https://genome.jgi.doe.gov

4
https://phytozome-next.jgi.doe.gov/
