# Supplementary material for: Long-day photoperiod and cool temperature induce flowering in cassava: Expression of signaling genes
Source: Front Plant Sci. 2022 Sep 16;13:973206. doi: 10.3389/fpls.2022.973206 (PMC9523484; doi:10.3389/fpls.2022.973206)
Supplement: Supplementary file 1 [file Data_Sheet_1.PDF]

Supplementary Figure 1A

Phytochrome Interacting Factor (PIF)

Manihot esculenta (Me) and
Ararbidopsis (At) homologs

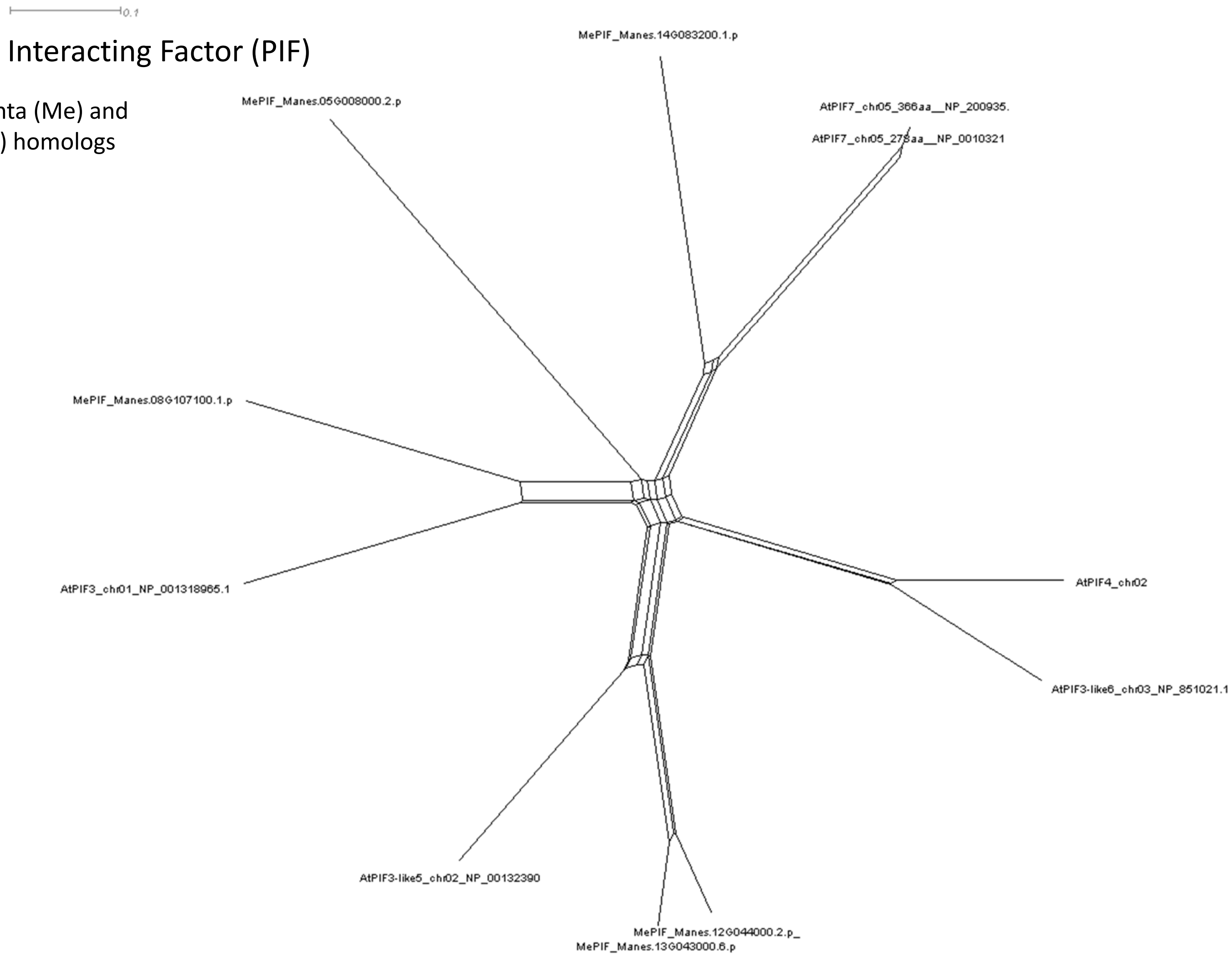

Supplementary Figure 1B

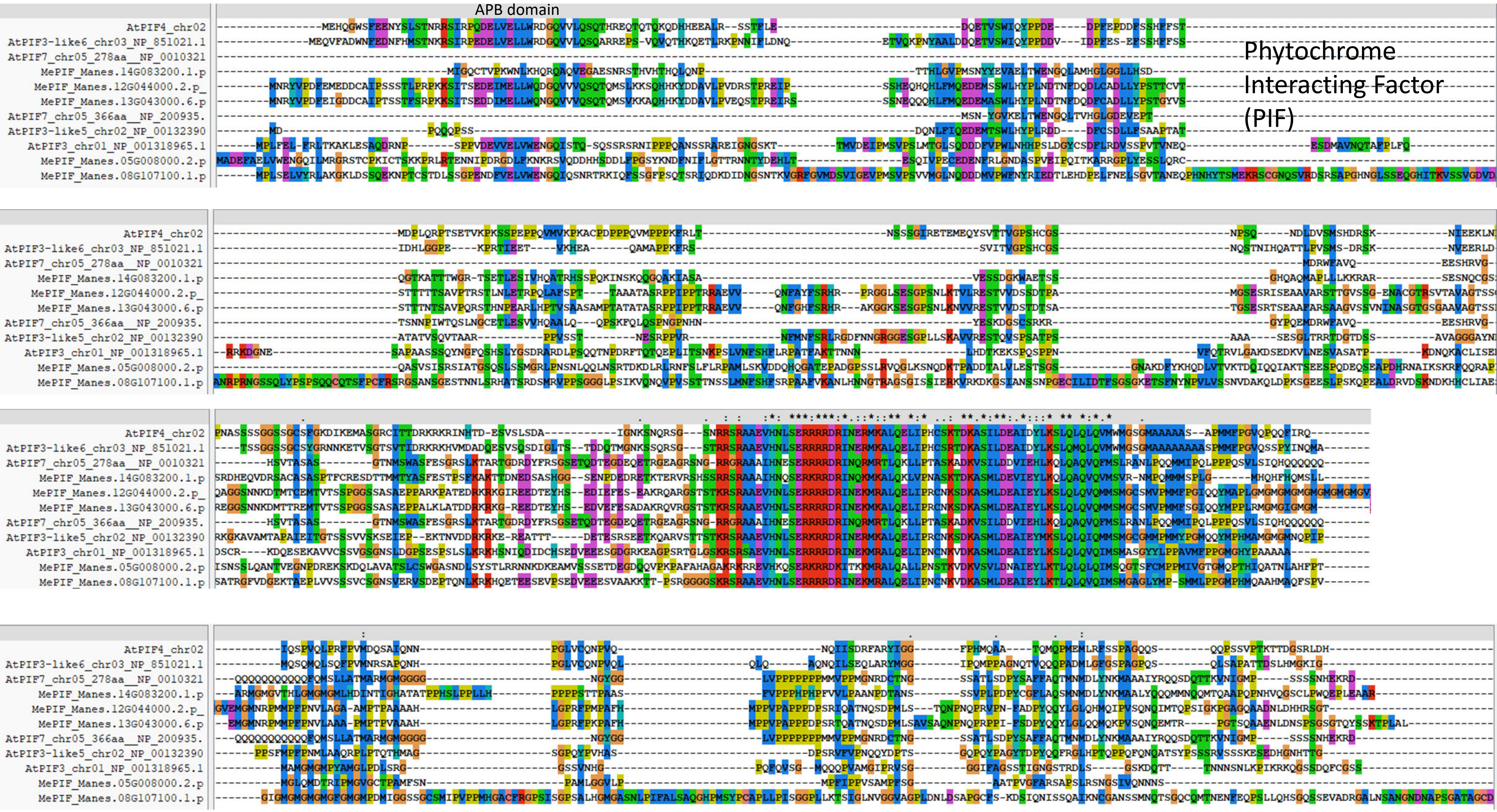

Supplementary Figure 2A

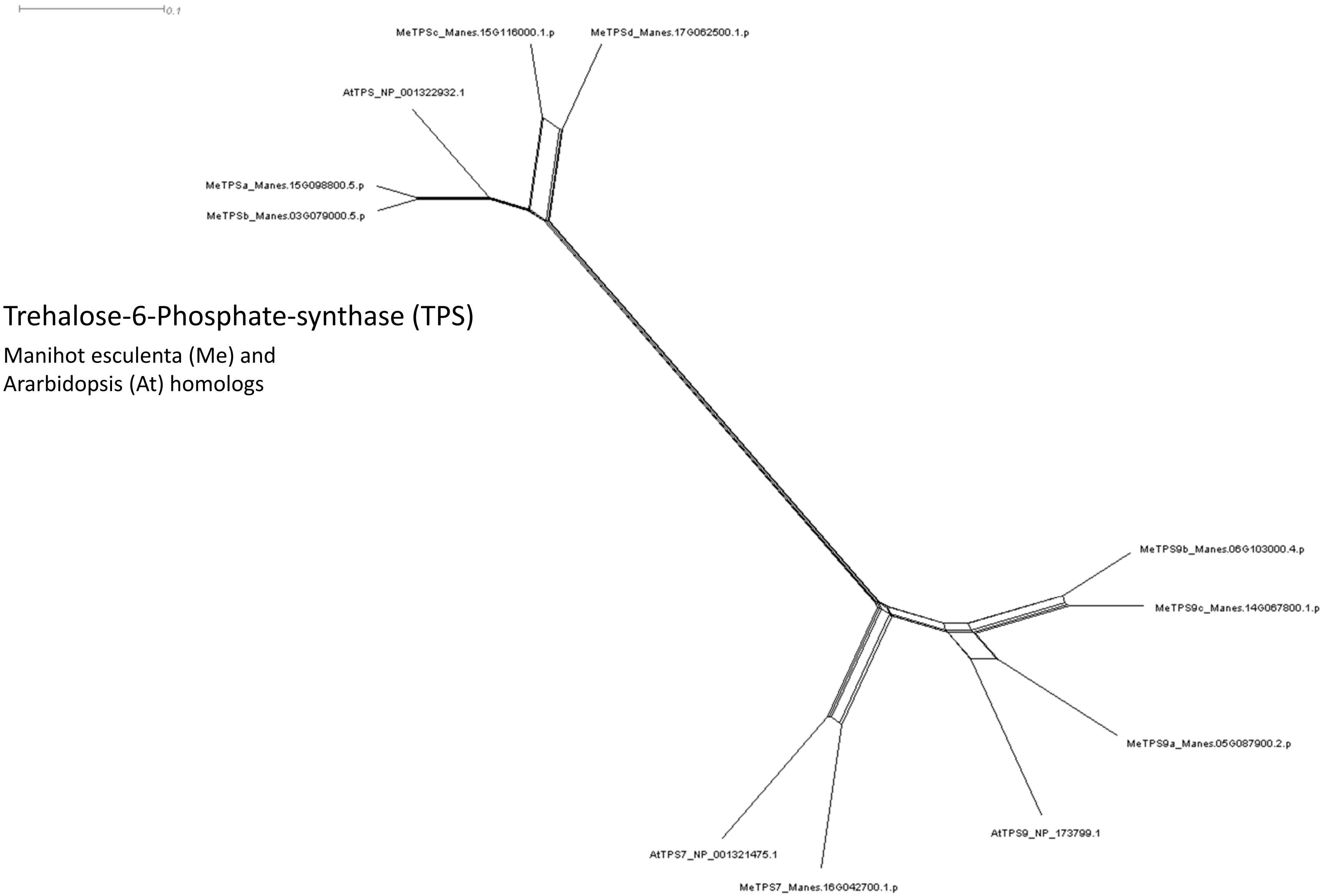

Trehalose-6-Phosphate-synthase (TPS)

Manihot esculenta (Me) and
Ararbidopsis (At) homologs

Supplementary Figure 2B

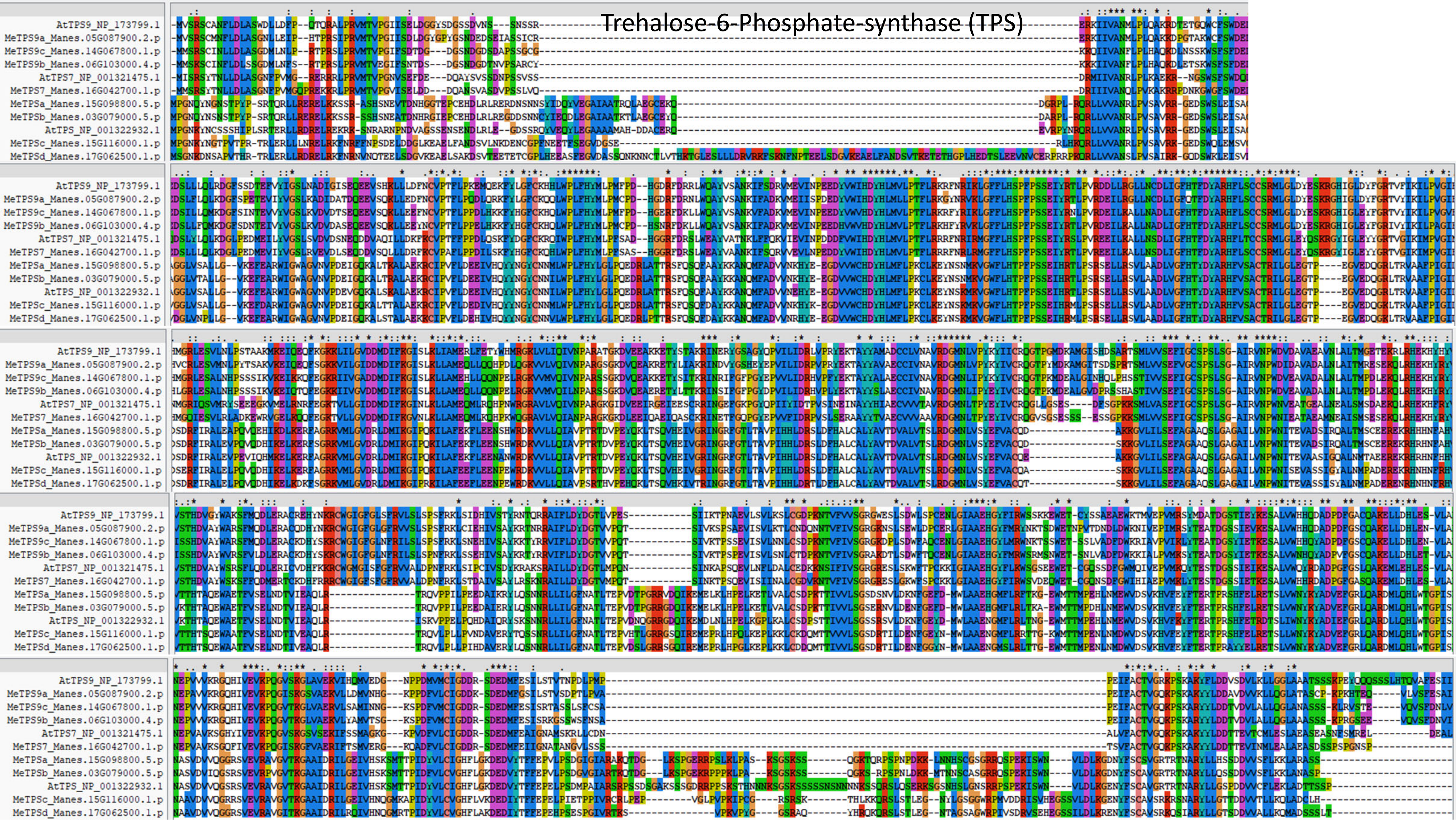

Supplementary Figure 3A

Squamosa Promotor Binding Protein-Like (SPL)

Manihot esculenta (Me) and
Ararbidopsis (At) homologs

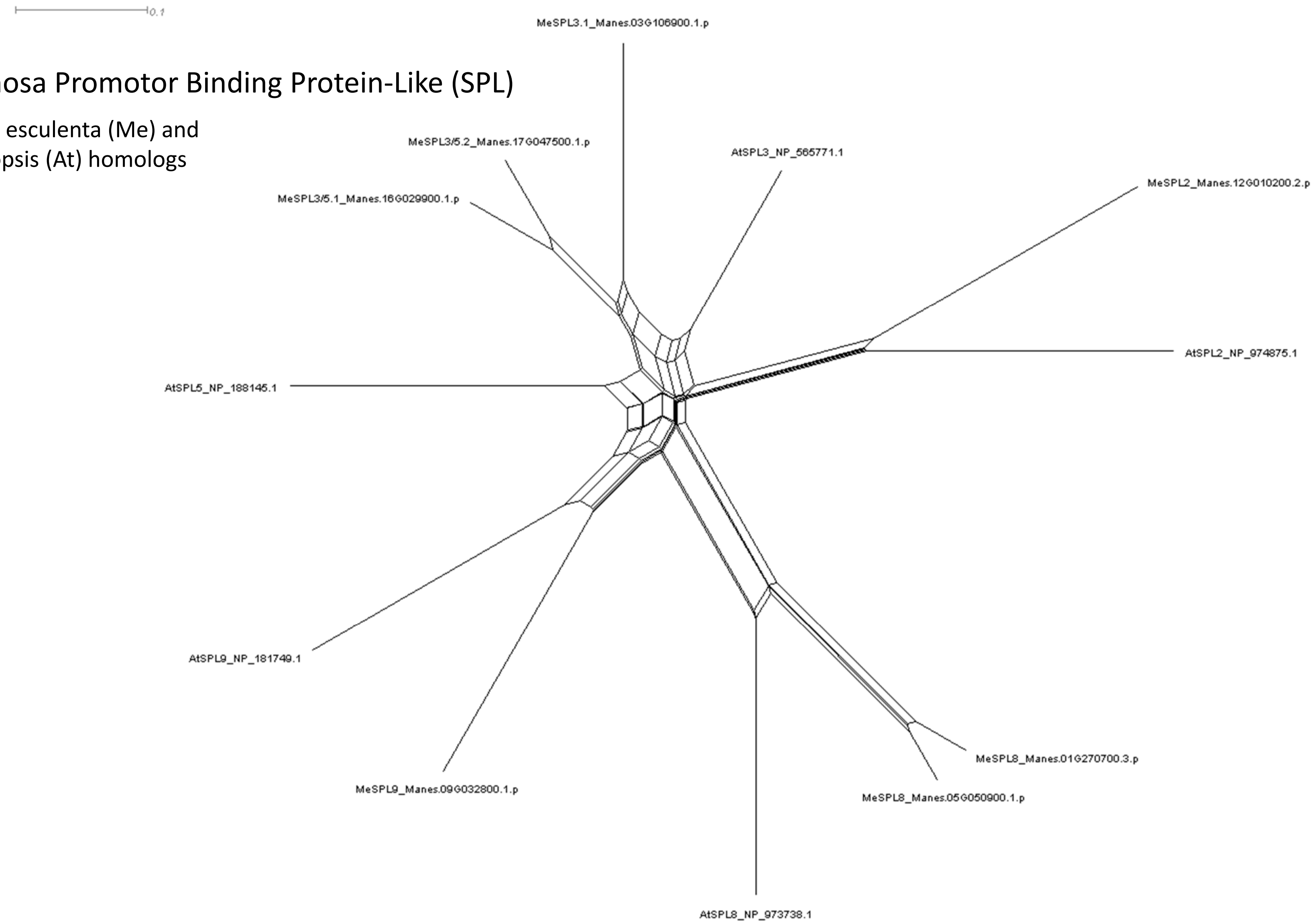

Supplementary Figure 3B

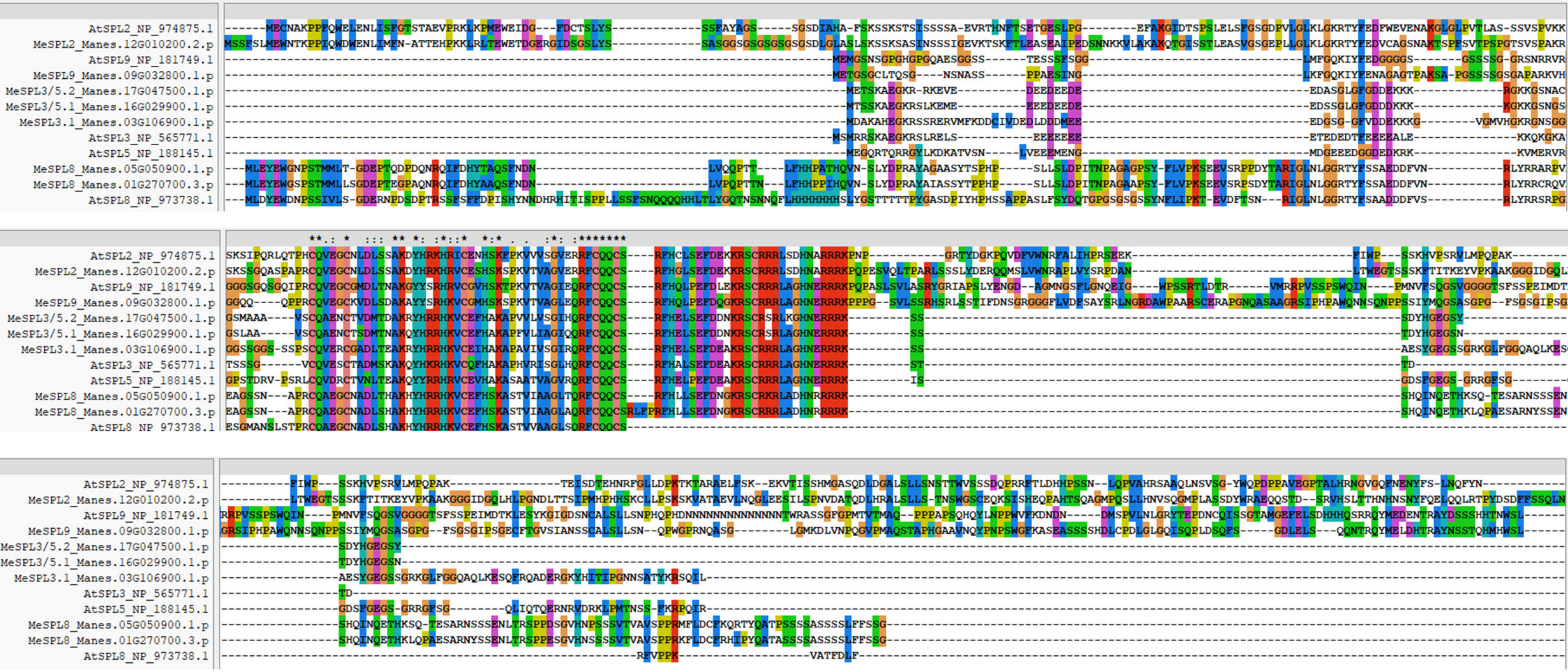

Squamosa Promotor Binding Protein-Like (SPL)
